# Supplementary material for: Aequorin as a Useful Calcium-Sensing Reporter in Candida albicans
Source: J Fungi (Basel). 2021 Apr 20;7(4):319. doi: 10.3390/jof7040319 (PMC8074299; doi:10.3390/jof7040319)
Supplement: Supplementary file 1 [file jof-07-00319-s001.zip › jof-1110504-supplementary.pdf]

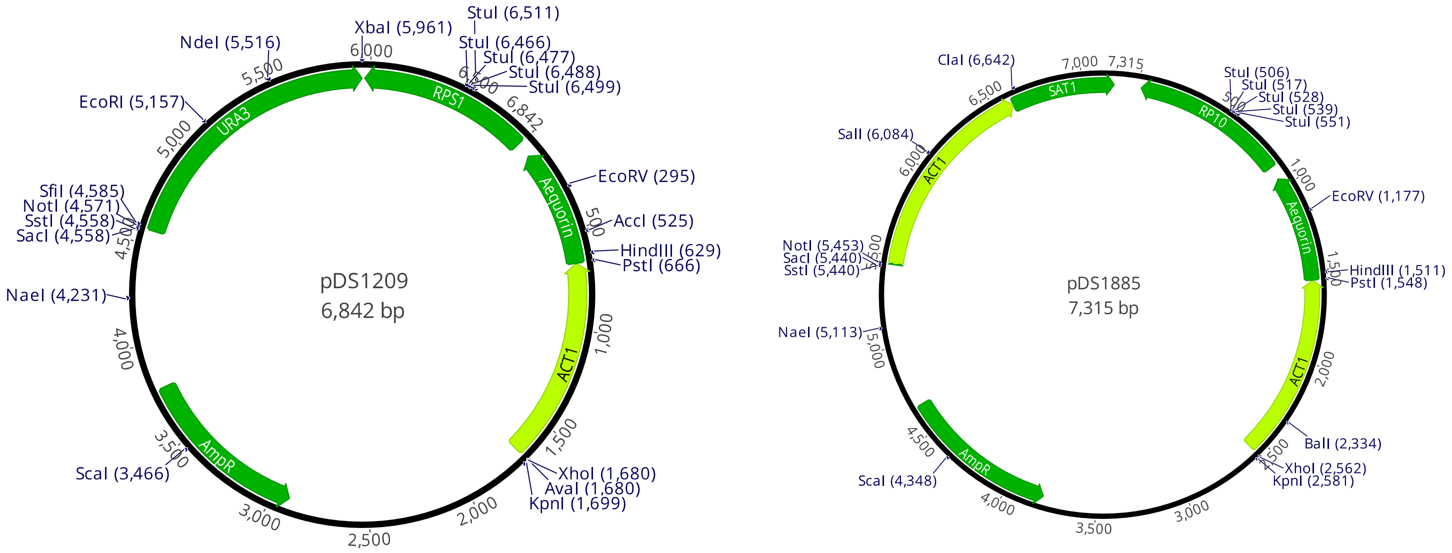

Figure S1: Plasmid map of aequorin expression vectors. Maps were obtained with the Geneious Prime software.

Table S1: Strains used in this study

**Isolate**	**Parent**	**Genotype**	**Reference**
SC5314	-	wild-type	[46]
CAF2-1	SC5314	*ura3Δ::imm434/URA3*	[46]
CAF4-2	CAF2-1	*ura3Δ::imm434/ura3Δ::imm434*	[46]
DSY3892	CAF4-2	*RPS1*::pDS1209	this study
DSY3999	CAF4-2	*VCX1*/*vcx1*Δ::hisG-*URA3*-hisG	this study
DSY4026	DSY3999	*VCX1*/*vcx1*Δ::hisG	this study
DSY4034	DSY4026	*vcx1Δ::hisG-URA3-hisG/* *vcx1Δ::hisG*	this study
DSY4058	DSY4034	*vcx1Δ::hisG/vcx1Δ::hisG*	this study
DSY4063	DSY4058	*vcx1Δ::hisG/vcx1Δ::hisG* *RPS1*::pDS1209	this study
JLR248	SC5314	*mid1*Δ::FRT/*mid1*Δ::FRT	[24]
JLR48	SC5314	*cch1*Δ::FRT/*cch1*Δ::FRT	[24]
JLR521	SC5314	*mid1*Δ::FRT/*mid1*Δ::FRT *cch1*Δ::FRT/*cch1*Δ::FRT	[24]
DSY4726	JLR248	*mid1*Δ::FRT/*mid1*Δ::FRT *RPS1*::pDS1885	this study
DSY4727	JLR48	*cch1*Δ::FRT/*cch1*Δ::FRT *RPS1*::pDS1885	this study
DSY4729	JLR521	*mid1*Δ::FRT/*mid1*Δ::FRT *cch1*Δ::FRT/*cch1*Δ::FRT *RPS1*::pDS1885	this study
DSY4730	SC5314	*RPS1*::pDS1885	This study

Table S2: List of primers used in this study

**Primer name**	**Primer sequence**
VCX-Kpn	TTTCCCTGGTACCAAGGATAAACTAAGAAT
VCX-BgB	TGGTAGATCTCTCATTCTCTGTTGGTTTG

VCX-PstB	ATAGATCTGCAGAAAGATCAAGCAAAGTTG
VCX-HindB	TAAAAGCTTTTGGTTTCCTAGTCAAAAACAG
AOQ-NHE	GCGCAAAGCTAGCTTAGGGGACAGCTCCACCGTAGAGCTTT
AOQ-PST	GCGCAACTGCAGAAAATGACAAGCAAACAATACTCAGTCAAGC
AOQ-5	GAGCCATTACATTGGATGAATGGAAAGCATACACCA
AOQ-3	CATTCATCCAATGTAATGGCTCCATTTTGATCTTTGTC
AOQ2-5	GAAGGATGGAAAAGATTGGCTTCCGAGGAATTGAAAAG
AOQ2-3	CTTTTCAATTCCTCGGAAGCCAATCTTTTCCATCCTTC
